# Dynamics of analyst forecasts and emergence of complexity: Role of information disparity

**DOI:** 10.1371/journal.pone.0177071

**Published:** 2017-05-12

**Authors:** Chansoo Kim, Daniel S. Kim, Kwangwon Ahn, M. Y. Choi

**Affiliations:** 1 Computational Economics Laboratory, Computational Science Research Center, Korea Institute of Science and Technology, Seoul 02792, Republic of Korea; 2 Department of Economics, Seoul National University, Seoul 08826, Republic of Korea; 3 HSBC Business School, Peking University, University Town, Nanshan District, Shenzhen 518055, P.R. China; 4 Moon Soul Graduate School of Future Strategy, Korea Advanced Institute of Science and Technology, Daejeon 34141, Republic of Korea; 5 Department of Physics and Astronomy and Center for Theoretical Physics, Seoul National University, Seoul 08826, Republic of Korea; Universidad Veracruzana, MEXICO

## Abstract

We report complex phenomena arising among financial analysts, who gather information and generate investment advice, and elucidate them with the help of a theoretical model. Understanding how analysts form their forecasts is important in better understanding the financial market. Carrying out big-data analysis of the analyst forecast data from I/B/E/S for nearly thirty years, we find skew distributions as evidence for emergence of complexity, and show how information asymmetry or disparity affects financial analysts’ forming their forecasts. Here regulations, information dissemination throughout a fiscal year, and interactions among financial analysts are regarded as the proxy for a lower level of information disparity. It is found that financial analysts with better access to information display contrasting behaviors: a few analysts become bolder and issue forecasts independent of other forecasts while the majority of analysts issue more accurate forecasts and flock to each other. Main body of our sample of optimistic forecasts fits a log-normal distribution, with the tail displaying a power law. Based on the Yule process, we propose a model for the dynamics of issuing forecasts, incorporating interactions between analysts. Explaining nicely empirical data on analyst forecasts, this provides an appealing instance of understanding social phenomena in the perspective of complex systems.

## Introduction

The twentieth century witnessed exponential growth in equity capital markets as more countries opened their securities exchanges and as the barrier to accessing capital markets became lower for the average household. In a rapidly growing capital market, one of the important players is the sell-side analyst, who provides valuable information on the issuers of securities, i.e., the firms. Sell-side analysts issue regular reports that summarize the future outlook of the business of a firm and forecast the performance, which includes forecasts on earnings per share (EPS), revenue, operating income, cash flow, and other accounting measures for both quarterly and annual performance. Analysts use financial statements, management communication [1], and detailed voluntary disclosure from the firms [2] as their information source for producing forecasts. Analyst forecasts on the accounting performance of firms are heavily followed and monitored by both company management and market participants, because accounting performance is the basis for forming expectations for future stock prices [3]. Due to their importance to the financial markets, analyst forecasts have long been of interest to scholars in finance and accounting.

So far, the vast majority of studies on analyst forecasts have focused on analyst consensus, which is either the mean or the median of non-stale analyst forecasts, i.e., existing but not too old (usually issued within last 90 days) forecasts [4–11]. Some other studies have focused on the volatility or dispersion of analyst forecasts [12–16]. It is known that analyst forecasts tend to be optimistic in the beginning and become lower towards the year-end [17].

There has been little interest in the outliers, or the tail distribution of analyst forecasts. While the location of the distribution is undoubtedly meaningful for understanding the views of sell-side analysts, the tails of the distribution also signal important messages. Regarding the analyst forecasts that deviate from the mass, Hong and collaborators [18, 19] found that inexperienced analysts are less likely to deviate from consensus with optimistic views relative to their more reputable counterparts. Put differently, this implies that if there is a positive outlier in analyst consensus, it may come from more reputable analysts. They argue that inexperienced or less reputable analysts are more liable to face problems with their jobs when their bold (more extreme) forecasts turn out to be wrong. In other words, unknown analysts are likely to be fired if they make forecasts that are different from others and turn out to be wrong. Recently, Du, Yu, and Yu [20] studied the most extreme outlier of the forecasts during the year, to find that the outlier triggers future earnings management by the company management.

In this work, we ask if complexity emerges in the collective behavior of analyst forecasts, particularly, among outliers of optimistic analyst forecasts. *Complexity* in general stands for large variability of the macroscopic behavior displayed by a many-particle system, consisting of many elements [21]. In such a system, appropriate interactions between elements can give rise to characteristic collective properties of the whole system, which may not be reduced directly to the properties of individual constituents. The appearance of such macroscopic behavior is thus called *emergence*. Even if (microscopic) interactions between elements are rather simple, the emergent (macroscopic) behavior can be very rich and diverse [22]. In our system, consisting of analysts as its elements, herding behavior emerges, as is evident from their forecasts. While actions of analysts do not necessarily delineate complexity, the whole system nevertheless exhibits such herding behavior and associated complexity. To investigate how forecasts form complex herding, we treat the forecasts as the product of small action rules (micromotives) of individual analysts and regard complexity as macrobehavior [23, 24].

We focus on optimistic forecasts because analyst optimism is a well-documented practice, and analysts are known to walk down their forecasts towards the end of a fiscal year [17]. Note that complexity is different from “analyst herding” reported in traditional accounting and finance literature [25]: while the latter refers to the tendency of analysts to issue forecasts that are simply closer to the mean of existing forecasts, complexity may arise from the tendency to issue forecasts distributed not only in the main body but also in the tail of optimistic forecasts.

Our study attempts to answer the following questions: Does complexity emerge in forecasts by major analysts (those issuing forecasts in the main body) or extreme analysts (those issuing forecasts in the tail of the forecast distribution); how are the forecasts tied to each other; what are the effects of the business cycle on analysts’ forecasts; does the emergent complexity change over time; what are the underlying factors for the changes; and is there any relation between the behavior of all analysts and that of extreme analysts? In this study, we employ complex system analysis to address these questions. Recently, more economists attempted to understand social phenomena by applying methods of statistical physics used for describing complexity [26–28]. Further, recent studies introduced statistical methodology to better explain the tail behavior of financial markets [29]. These methods allow us to characterize economic systems and help us better understand their tail behavior.

To understand the behavior of analyst forecasts, we here consider the Yule process, described by a master equation. This turns out to allow for the emergence of skew distributions, including power-law and log-normal distributions. Power-law behavior, a prominent statistical signature for complexity [30, 31], has been recognized also in complex economic systems. The power-law exponent is found to be an important and even universal factor in characterizing financial markets, such as stock markets and foreign exchange markets [29, 32–35]. Increasing awareness of the power law has recently spurred attempts to explain emergent characteristics of various economic and social phenomena of complexity [36]. We thus probe the emergence of power-law as well as log-normal distributions, which serves as a key evidence of complexity, in analyst forecasts on EPS of publicly traded US firms.

In the following sections, details of the empirical evidence, which is based on the big-data analysis, are presented. We then provide a theoretical framework, incorporating interactions between analysts. The proposed model is shown to explain well the complexity observed in the empirical data on analyst forecasts. Finally, a summary with discussion is given.

## Empirical evidence

In this section we present our empirical results based on the analyst forecast error, which measures the difference between the analyst forecast on EPS and the actual EPS outcome. We scale the difference by the stock price, to normalize the difference across sample firms.

Analyst forecast data are obtained from the Institutional Brokers’ Estimate System (I/B/E/S), which reports the EPS forecast value (VALUE) *z*, date (ANNDATS), and time (ANNTIMS) of the forecast, along with the brokerage house (ESTIMATOR) and analyst (ANALYS). I/B/E/S also reports the actual EPS performance (ACTUAL) *a* of the fiscal year. Our main variable of analysis is the analyst forecast error

$$\begin{array}{c} x\equiv \frac{z-a}{S}, \end{array}$$ (1)

which is scaled by the stock price *S* on the previous trading day. It turns out that a vast majority of forecast values are greater than the actual EPS performance, reflecting the optimistic views of most analysts. Accordingly, in this study, we take only observations with positive forecast errors, to focus on optimistic forecasts, and perform big-data analysis on the I/B/E/S data spanning nearly thirty years.

Our analysis manifests that the analyst forecast errors display two distinct distributional patterns: the main body and the tail. In the main body part, defined as forecast errors greater than zero but smaller than the 95th percentile value, forecast errors follow a log-normal distribution; in the tail part, defined as those larger than the 95th percentile, forecast errors follow a power-law distribution, where collective behavior among extreme forecast errors is expected [37]. For the tail, we use precisely 95.0th to 99.7th percentiles of observations to account for data errors as well as penny stock cases after the 99.8th percentile.

The distribution of forecast errors in the main body is found to be skewed, and turns out to fit the log-normal distribution, specified by the probability density function (PDF):

$$\begin{array}{c} f\left(x\right)=\frac{1}{\sqrt{2\pi }\sigma x}\exp\left[-\frac{{\left(\ln x-\mu \right)}^{2}}{2\sigma^{2}}\right]. \end{array}$$ (2)

**Fig 1:**
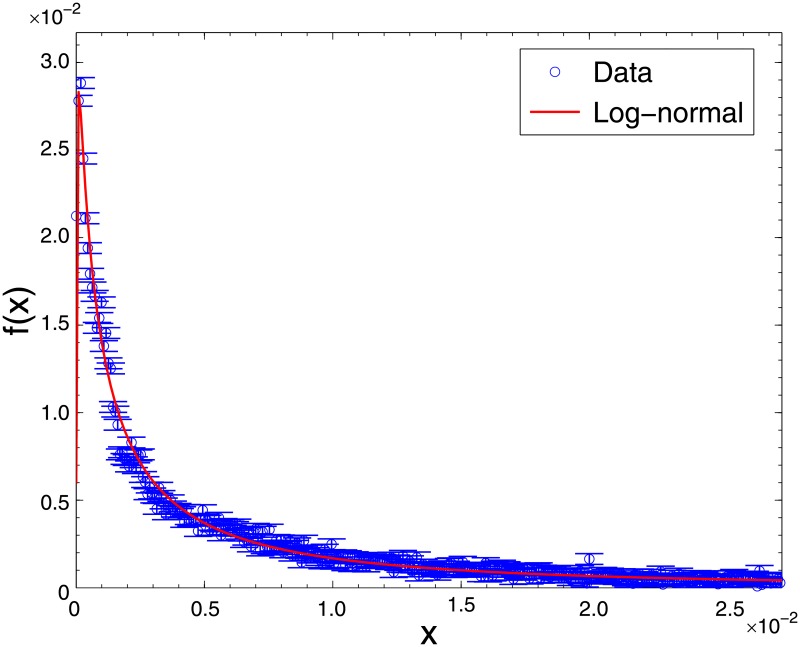
Log-normal fit of the main body part of forecast errors. We fit the main body part of analyst forecast errors to the log-normal distribution. (The figure is based on non-stale analyst forecast errors on the last trading day of 1993; main body parts from other calendar years fit log-normal distributions as well.) We define the main body part as the bottom 95 percent of analyst forecast errors (129202 observations in 1993). The red line is the fitted log-normal density while blue circles represent the data points. Error bars have been estimated from the deviation of the data. The fitted parameter values are: location parameter *μ* = −5.46 and scale parameter *σ* = 1.94.

In fitting our sample, we first construct histograms with optimal binwidth for the forecasts errors in the main body. We then use the maximum likelihood estimation (MLE) method, to estimate the mean and deviation of the log-normal distribution within the 95% confidence interval. Fig 1 illustrates the resulting log-normal fit of our sample for calendar year 1993 with mean *μ* = −5.46 and deviation *σ* = 1.94. The forecast errors for other years in our sample, left out for brevity, also fit log-normal distribution very closely.

To disclose the detailed behavior of the tail, we consider the cumulative distribution function (CDF) *F*(*x*), which is given by the integral of the PDF from −∞ to *x*. The power-law behavior described by the CDF [29, 37]

$$\begin{array}{c} 1-F\left(x\right)∼x^{-\alpha }, \end{array}$$ (3)

corresponds to the PDF *f*(*x*) = *dF*(*x*)/*dx* ∼ *x*^ − (1+*α*)^.

**Fig 2:**
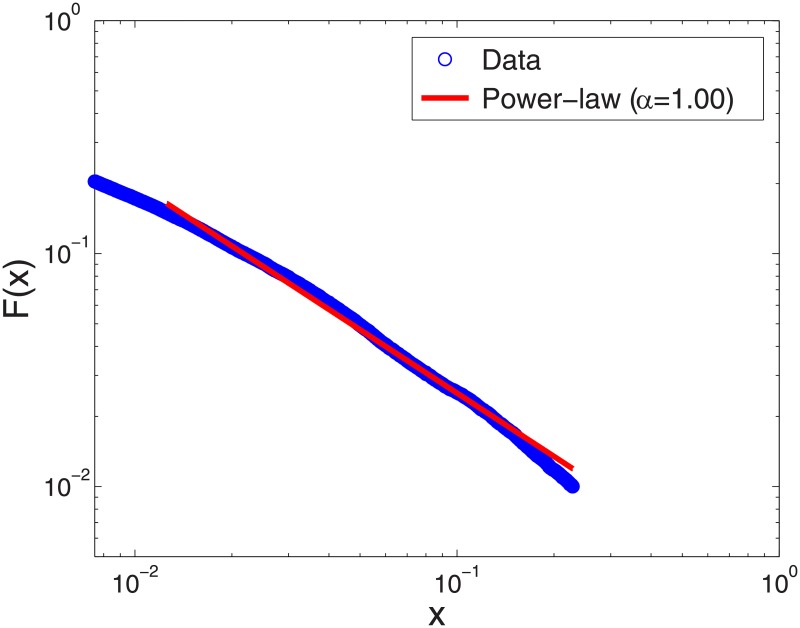
Power-law fit of the tail of forecast errors. We fit the tail part of analyst forecast errors to the power-law distribution. (The figure is based on non-stale analyst forecast errors on the last trading day of 1993; tail parts from other calendar years fit power-law distributions as well). Since the power-law distribution in Eq (3) implies a linear relationship in the logarithmic scale, we use ordinary least squares (OLS) to fit the empirical cumulative distribution. The red line depicts the fitted power-law distribution while blue circles represent the data points. In 1993, the power-law exponent approximates unity (*α* ≈ 1), which indicates that analyst forecast errors follow Zipf’s law.

Since Eq (3) exhibits a linear relationship in the logarithmic scale, we use the ordinary least squares (OLS) method to fit the power-law PDF *f*(*x*). Constructing an empirical cumulative distribution from the data on forecast errors in the tail, we use OLS regression to estimate the exponent *α* of the power-law distribution. It minimizes the errors between *F*(*x*) and the empirical distribution. From Fig 2, we find that the tail of analyst forecast errors indeed fits the power-law distribution, implying collective behavior among forecast errors. In year 1993, with 16508 forecast errors in the tail, the estimated power-law exponent is *α* = 1.00 ± 0.01.

The power-law behavior of forecast errors suggests that analyst forecasts are based on the “principle of least effort” [38, 39]. Specifically, the power-law exponent unity, following Zipf’s law, implies that information seeking analysts tend to use the most convenient search method and such extremely optimistic analysts behave collectively. In Theoretical Framework section, emergence of such skew distributions as log-normal and power-law distributions is explained by means of controlled growth processes [40].

### Yearly pattern in extreme forecast errors

**Fig 3:**
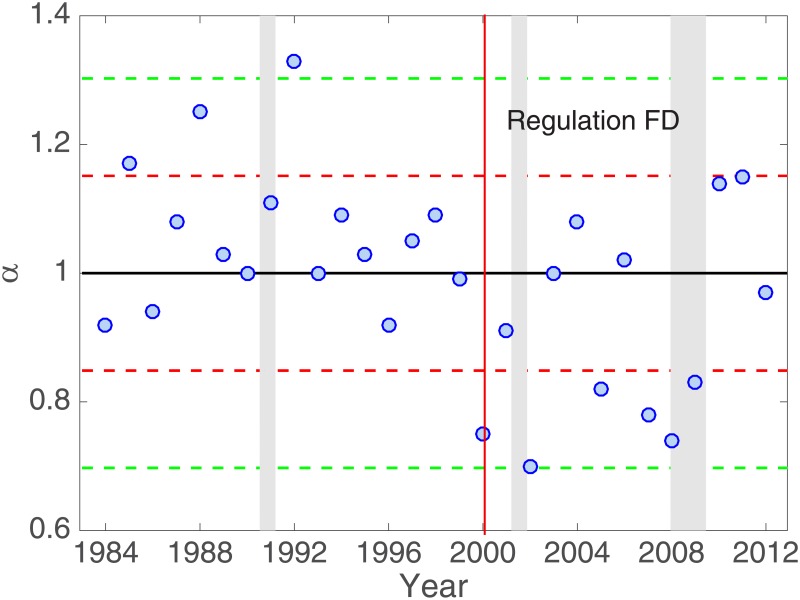
Annual values of the power-law exponent. Blue dots represent annual estimates of the power-law exponent *α* from year 1984 to 2012. The annual value of the exponent is estimated by fitting all forecast errors in each calendar year. The black horizontal line represents the power-law exponent of unity (*α* = 1), which corresponds to Zipf’s law, while two red/green dashed lines indicate one/two standard deviations away from Zipf’s law. The vertical line designates year 2000, the year of “Regulation Fair Disclosure (FD)”, while the shaded periods correspond to NBER recessions.

For each year in our sample period, 1984 to 2012, we estimate the power-law exponent *α* for analyst forecast errors and obtain the mean value 1.00 with standard deviation 0.15. From *α* ≈ 1, we recognize that the tails of the analyst forecast errors follow Zipf’s law [38] in the whole (sample) period, as presented in Fig 3.

It is of interest that while most years have *α* close to unity, in certain years *α* deviates from unity by more than one standard deviation. It takes values lower by more than one standard deviation in years 2000, 2002, 2005, 2007, 2008, and 2009. The lower the value of *α* is, the thicker the tail distribution is. This implies that collective behavior is weaker in the above mentioned years. In contrast, collective behavior is stronger in years with *α* higher than unity by more than one standard deviation: 1985, 1988, and 1992.

Comparing Fig 3 with recession data from National Bureau of Economic Research (NBER), we notice that the years when *α* deviates by more than one standard deviation overlap largely with the recession period in the U.S. This indicates that analysts, facing economic recessions, tend to either follow extreme opinions (higher *α*) or reject them (lower *α*).

It is also broadly accepted that information asymmetry or disparity is more severe during a recession as the prospects of the economy are more uncertain, and as a consequence, analyst forecasts are more dispersed than in other periods [41, 42]. Information disparity in the market is higher just before an economic recession, as it is in general more difficult to forecast the future before huge changes take place. Thus the exponent *α* deviating from unity by more than one standard deviation is consistent with our intuition that extreme analyst forecasts should behave differently during a recession compared with a normal period. The relationship between the recession and the S&P500 index (https://research.stlouisfed.org/fred2/series/SP500) indeed confirms this intuition [43, 44].

Yet, it is not straightforward to explain the discrepancy between higher and lower values of *α* in different recession periods. The year separating the two cases provides us with an important clue. Those values deviating largely from unity display sharp contrast in the general trend before and after 2000: They are higher than the normal values before 2000, but are lower during and after 2000. We propose that this is the evidence of a structural break in information disparity in the U.S. markets. Specifically, in October 2000, the Securities Exchange Commission (SEC) passed “Regulation Fair Disclosure (FD)”, which requires that all information be fairly disseminated to the market [45]. This regulation is intended to reduce information disparity in the financial market by allowing equal access to firm information for all market participants. Prior to Regulation FD, companies could disclose firm information selectively to only some of the analysts and participants in the market; the regulation prohibits such selective disclosure practice. While there are arguments about the actual outcome of the regulation, some researchers do find improvements in the information environment after the regulation [46, 47].

When there is higher information disparity, namely, when only some analysts can access information, it is plausible to expect that those without the information will follow the opinions of those with the information. This mimicking behavior will cause some analysts to issue forecasts that are closer to extreme forecasts of other sell-side analysts, usually more reputable ones, giving rise to collective behavior among the outliers of analyst forecasts. Such collective behavior resulted in higher values of *α* during recession periods in the years before Regulation FD. However, when this information disparity weakens in the market, all financial analysts share the same set of information and there is no need for the sell-side analysts to follow extreme analyst forecasts. This results in lower values in the years of post-Regulation FD. It was also claimed that the positive effects of Regulation FD are more pronounced for firms that suffer from more information disparity (illiquid firms) [46], which is consistent with our findings. We thus conclude that Regulation FD made the information environment more effective during recessions.

We now test formally our conjecture from Fig 3. We choose the analyst forecast dispersion, defined as the standard deviation of analyst forecasts, as the proxy for information disparity. Unlike existing studies adopting the analyst forecast dispersion as the proxy for information asymmetry at the firm level, we consider the information disparity of the whole market. As such, we develop a market-wide analyst forecast dispersion for each sample year. We first scale the forecast dispersion by analyst consensus (mean of analyst forecasts) for each firm in our sample. The market-wide forecast dispersion is then given by the means of the scaled forecast dispersions of all companies in our sample. We also adopt the forecaster bias [48] as the proxy for economic uncertainty, which is in literature found high during the periods of economic downturn. We use the economic uncertainty as the continuous alternative to the NBER recession indicator; however, results using the NBER recession indicator are qualitatively similar. Table 1 presents regression results of information disparity and the power-law exponent.

**Table 1:** Regressions of information disparity and the power-law exponent. Models (1) and (2) test the association of information disparity and economic uncertainty; Models (3) and (4) test how the power-law exponent *α* deviates from unity due to information disparity. Models (5) and (6) test how collective behavior changed after the Regulation FD was introduced. *R* takes the value unity for years after Regulation FD was introduced and zero otherwise, *I* is the proxy for information disparity measured by the scaled forecast dispersion of the market, *E* is the economic uncertainty measure from Ref. [48] and *c* is constant. The numbers in parentheses are *t*-statistics calculated from heteroskedasticity and autocorrelation consistent standard errors according to Newey and West [49]. ** and *** represent significance at 5% and 1% levels, respectively.

Model	(1)	(2)	(3)	(4)	(5)	(6)
	*I*	*I*	|*α* − 1|	(*α* − 1)^2^	*α*	*α*
*R*		−0.148*** (−8.62)			−0.147*** (−5.40)	0.00601 (0.09)
*I*			0.841*** (3.29)	0.196*** (2.45)		0.832*** (2.64)
*E*	0.0438** (2.26)	0.0544*** (4.62)				
*E* ⋅ *R*		0.402*** (9.73)				
*R* ⋅ *I*						−1.499*** (−3.00)
*c*	0.0800*** (6.25)	0.0653*** (9.28)	0.0294 (1.30)	0.00205 (0.28)	1.062*** (89.50)	0.981*** (30.83)
Observations	29	29	29	29	29	29
Adjusted *r*^2^	0.077	0.394	0.025	0.002	0.215	0.173

We first test whether information disparity in the stock market measured by the dispersion in analyst forecasts is statistically associated with economic uncertainty and whether the association has changed significantly since the Regulation FD. Our empirical models describe how information disparity *I* is related to economic uncertainty *E*:

$$\begin{array}{c} I=c_{1}+\beta_{1}E+\varepsilon_{1} \end{array}$$ (4)

and

$$\begin{array}{c} I=c_{2}+\beta_{2}R+\beta_{3}E+\beta_{4}R\cdot E+\varepsilon_{2} \end{array}$$ (5)

with appropriate constants *c*_*i*_ and *β*_*i*_, where *R* takes into account Regulation FD (*R* = 0 and 1 for calendar years before and after Regulation FD is introduced, respectively), *R* ⋅ *E* represents the interaction of *R* and *E*, and *ε*_*i*_ denotes the error term.

In Model (1), the analyst forecast dispersion is significantly positively associated with economic uncertainty, supporting our argument that a higher level of economic uncertainty leads to a higher level of information disparity in the market. Further, the significantly negative loading on Regulation FD in Model (2) indicates that information disparity in the stock market has generally decreased since Regulation FD put in place. Economic uncertainty still seems to positively influence information disparity in general. Interestingly, the loading on the interaction term of economic uncertainty and regulation is significant and positive. This indicates that economic uncertainty is more closely linked to information disparity in the stock market after the introduction of Regulation FD. This is rather intuitive when we understand the potential sources of information disparity. Specifically, information disparity is caused by at least two factors: 1) imperfect information disclosure and 2) uncertainty in the market. The Regulation FD was introduced to reduce information disparity in the stock market by forcing companies to share information equally among market participants. Thus the Regulation FD effectively eliminates the first source of information disparity, making uncertainty more influential in the stock market.

Models (3) to (6) test how information disparity affects estimates of the power-law exponent *α*. In Fig 3, we observe that the power-law exponent deviates from unity, corresponding to Zipf’s law, during recessions. Models (3) and (4) employ the absolute deviation from unity, |*α* − 1|, and the squared deviation from unity, (*α* − 1)^2^, respectively, as the dependent variables and are thus specified by

$$\begin{array}{c} \left|\alpha -1\right|=c_{3}+\beta_{5}I+\varepsilon_{3} \end{array}$$ (6)

and

$$\begin{array}{c} {\left(\alpha -1\right)}^{2}=c_{4}+\beta_{6}I+\varepsilon_{4}, \end{array}$$ (7)

respectively. As evident from Table 1, in years with higher levels of information disparity, the power-law exponent deviates more from unity, either upward or downward.

In Models (5) and (6) the level of the power-law exponent is adopted as the dependent variable in the following way:

$$\begin{array}{c} \alpha =c_{5}+\beta_{7}R+\varepsilon_{5} \end{array}$$ (8)

and

$$\begin{array}{c} \alpha =c_{6}+\beta_{8}R+\beta_{9}I+\beta_{10}R\cdot I+\varepsilon_{6}. \end{array}$$ (9)

In Model (5), where only the Regulation FD is used as the independent variable, it appears that the power-law exponent generally becomes smaller after the introduction of Regulation FD. However, in Model (6), one can clearly see that this is not the case. The Regulation FD itself does not affect the power-law exponent. Model (6) suggests that the power-law exponent is unconditionally close to unity, indicated by the constant term 0.98. Furthermore, the power-law exponent is significantly positively and negatively affected by information disparity before and after the Regulation FD, respectively (specifically, a unit increase in information disparity will lead to the increase and decrease in the power-law exponent by 0.83 and 0.67, respectively). These results are consistent with our observation in Fig 3 that analyst forecast errors follow Zipf’s Law in ordinary years. They are also consistent with our observation during recessions that analysts tend to herd more in the tail before Regulation FD and more to the consensus after Regulation FD.

### Monthly pattern in extreme forecast errors

**Fig 4:**
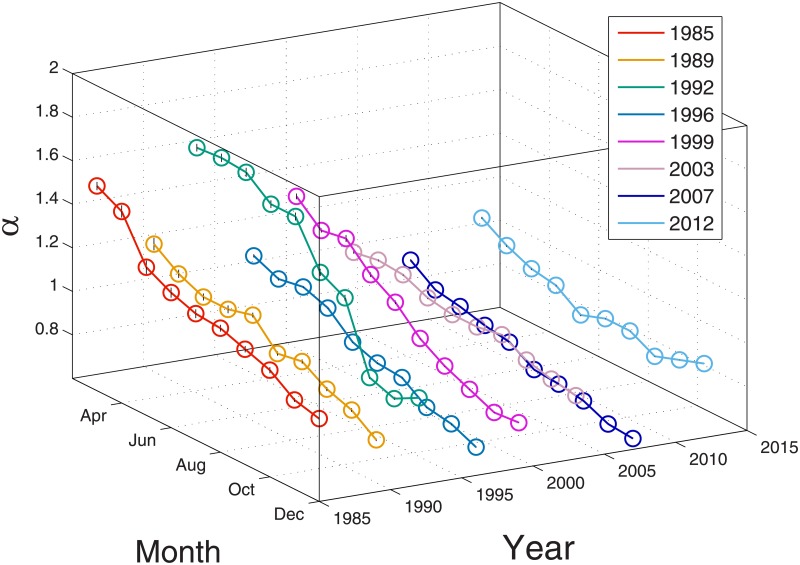
Monthly variations of the power-law exponent by year. Shown are the data in calendar years 1985, 1989, 1992, 1996, 1999, 2003, 2007, and 2012; these sample years have been chosen for the sake of brevity, but the trends in other calendar years are largely similar to those shown in this figure. The colored circles represent the monthly estimates of the power-law exponent from March to December in each given year, with error bars estimated from the power-law fit. Lines connecting circles are merely guides to the eye. There appears a clear descending pattern of the power-law exponent within a year. Namely, the power-law exponent tends to decrease towards the year-end.

Observing that *α* can take different values over time, we next investigate whether there is any time-series pattern in *α* within a given year. Since we use analyst forecasts on annual EPS, which will be realized after the fiscal year end, analysts forecasts issued earlier in the year suffer from higher uncertainty than those issued later in the year. Thus the change of the power-law exponent within a year reflects the change in collective behavior of analysts due to the change in the informational environment. We first plot in Fig 4 monthly changes of *α* in eight sample years, which are selected randomly. It is observed that the majority shows a clear descending pattern as it approaches the end of a year. (Data for all other years also exhibit qualitatively the same results, albeit not shown for the sake of readability.) Referring to our previous explanation, the decreasing value of *α* implies that the collective behavior becomes weaker.

This leads to a counterfactual conclusion: extreme analysts tend to flock less as the year-end approaches, i.e., as information disparity reduces closer to the year-end, there are fewer uncertainties regarding the outcome of the fiscal year. Analysts have less reason to follow blindly extreme forecasts by other analysts because all analysts are provided with richer information about the firms. This result is consistent with our finding in the annual analysis in the previous section. On the other hand, it is conceivable that better information should lead analysts to issue similar forecasts closer to the actual outcome.

**Fig 5:**
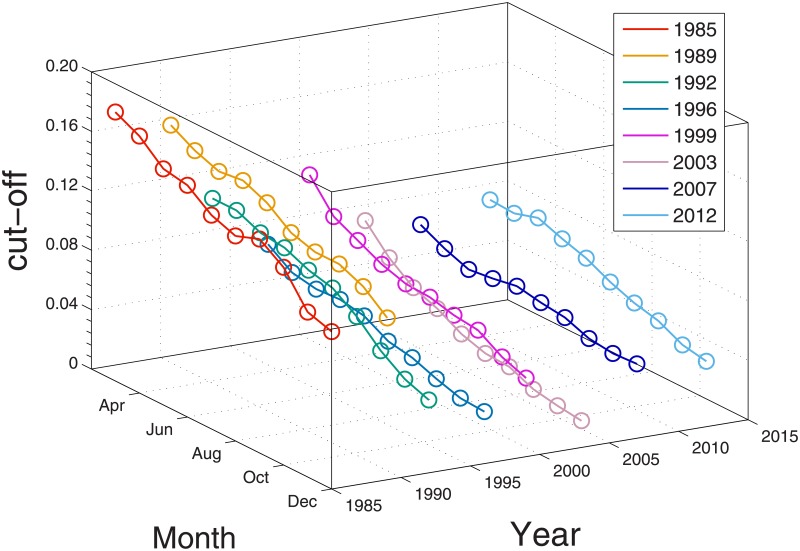
Monthly cut-off points by year. For comparison, the same sample years as those in Fig 4 have been selected. We show the cut-off points for the tail part of forecast errors (95th percentile of the observations) from March to December in each given year. The descending trend of cut-off points manifests that the forecast errors converge to zero towards the year-end, which implies that analysts are becoming more precise in their forecasts.

A key to resolve this discrepancy is to check the cut-off point (95th percentile) of the tail as our findings for the collective behavior of analyst forecast errors come from the tail. Fig 5 shows the monthly cut-off points in each year. We clearly observe that the cut-off point decreases as it gets closer to the year-end. This indicates that the distribution of analyst forecast errors clusters more around zero towards the year-end.

In other words, we see that analysts lose their collective behavior in the tail, and at the same time they herd toward the center as time goes by. Overall, Figs 4 and 5 suggest that analyst forecasts clearly become less biased closer to the year-end and analysts are less likely to follow blindly extreme forecasts.

### Monthly pattern in non-extreme forecast errors

As shown in Fig 1, forecast errors in the main body form a log-normal distribution. To probe the monthly pattern in non-extreme forecast errors, we fit the monthly data to the log-normal distribution for each month in calendar year 1993 and examine how the distribution evolves in time. Investigation of such monthly dynamics of the main body may also help substantiate the monthly pattern in extreme forecast errors as the pattern of the main body should relate with that of the tail.

Note that a log-normal distribution has two parameters: location parameter *μ* corresponding to the mean and scale parameter *σ* measuring the deviation. If the location parameter is smaller, the bulk of the distribution locates closer to zero whereas the scale parameter affects the skewness as well as thickness of the tail. The larger the scale parameter, the more positively skewed and fatter the tail. Namely, the tail is pulled more in the positive direction and becomes thicker [50] in the main body.

**Fig 6:**
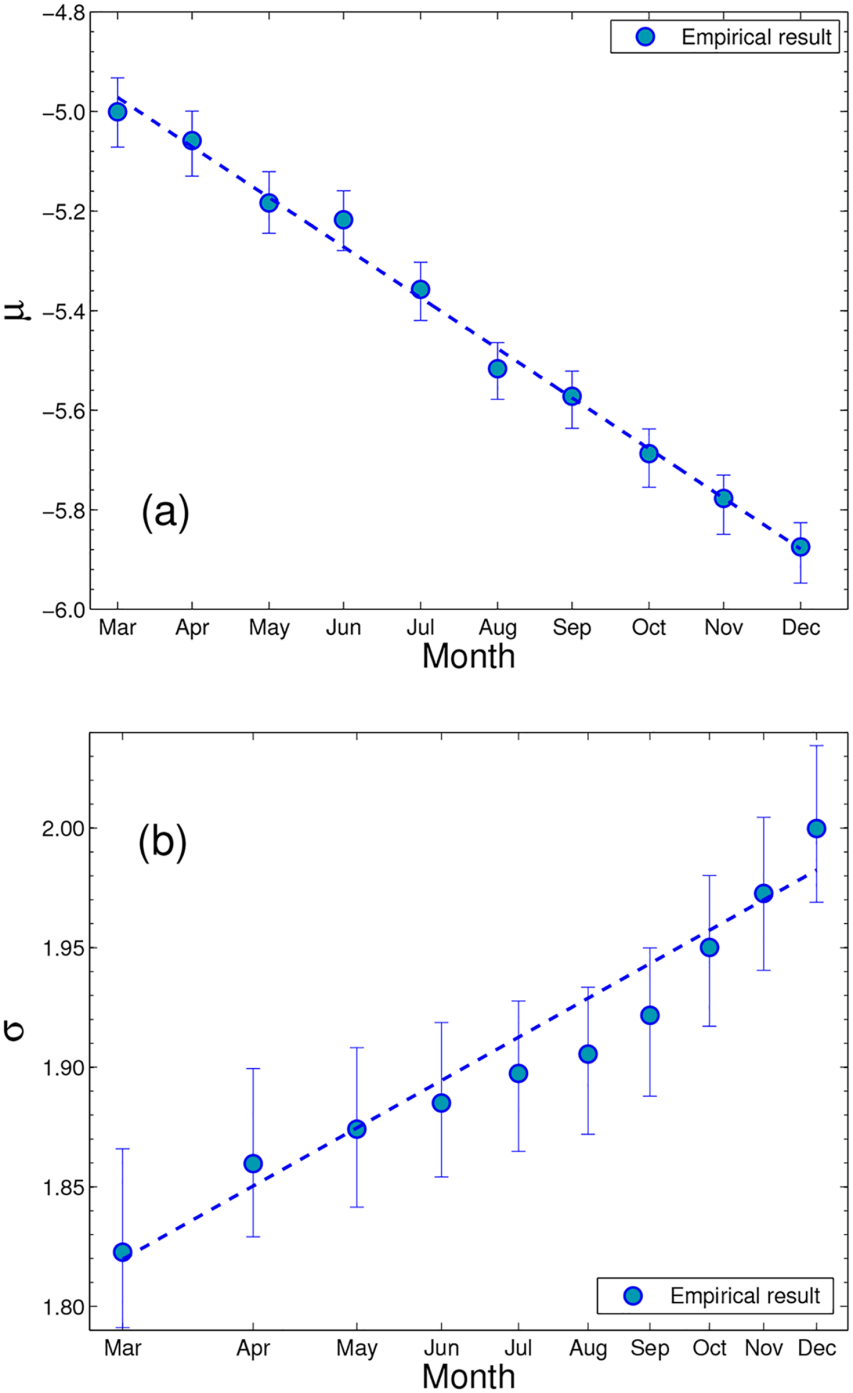
Monthly evolution of (a) the location parameter *μ* and (b) the scale parameter *σ*. This figure presents the monthly changes in *μ* and *σ* of the log-normal fit of the main body part of forecast errors in 1993. As predicted by the model, *μ* is linear in time and *σ* is linear with respect to the square root of time (note that the scale of the horizontal axis is given in the square root of time). Blue dots in (a) and (b) are the estimates of the location and the scale parameters, respectively; dashed lines in (a) and (b) have the slopes −0.0032 and 0.0148, respectively, per day.

Fig 6 exhibits the monthly change of the two parameters. It is observed that *μ* reduces linearly with time *t* and that *σ* increases in proportion to the square root of time: *μ* = *At* + *μ*_0_ and $\sigma =B\sqrt{t}+\sigma_{0}$ with the slopes *A* = −(3.2 ± 0.5) × 10^−3^ and *B* = (1.5 ± 0.6) × 10^−2^ from March to December (with *t* in units of day). This indicates that the distribution of the main body moves toward smaller values in each month while becoming fatter in the tail.

## Theoretical framework

The fitted distributions can be understood conveniently by the master equation description of the Yule process, originally proposed to explain growth of particle size [40, 51]. Making use of the similarities between the particle growth and analysts’ behavior, this approach explains resulting skew distributions of analyst forecasts, including log-normal as well as power-law distributions.

In the master equation, the transition rate is constructed from analyst’s forecast updates and we obtain the evolution equation for the distribution of analyst forecasts. Brown and collaborators [52] suggested important information sources or considerations for analysts’ earnings forecasts, which are, in the order of importance, industry knowledge, communication with management, earnings conference calls, management earnings guidance, quality or reputation of management, recent earnings performance, recent Form 10-K (annual report of a company’s financial performance required by the SEC) or Form 10-Q (quarterly report), primary research, other analysts’ earnings forecasts, own stock recommendations, and recent stock price performance.

While earnings conference calls, management earnings guidance, quality or reputation of management, recent earnings performance, recent Form 10-K or Form 10-Q, and own stock recommendations are available only sporadically or quarterly over time, other factors can be updated more often. In this view, if an analyst does not update the earnings forecast on a particular day, that analyst is counted issuing the same earnings forecast as the previous day. Namely, according to the Bayesian inference, we assume that an analyst, when no new information is available, would not issue a new forecast.

We thereby consider the forecasts to be evolving elements and the forecast errors to form a distribution evolving according to forecast updates, which reveals analysts’ behavior. Specifically, an analyst’s forecast error is viewed as the “size” of the element. The size *x* grows or diminishes, namely, the forecast error *x* changes to *x*′, when an analyst updates a forecast upon the arrival of new information; we call this *an event*.

It is expected that the amount of growth, Δ*x* ≡ *x*′ − *x*, depends on the present value *x*. Based on the Yule process, we define that Δ*x* is proportional to *x*: Δ*x* = *bx*, and write the transition rate associated with an event in the form

$$\begin{array}{c} \omega \left(x\to x^{\prime }\right)=\lambda \delta \left[x^{\prime }-\left(1+b\right)x\right], \end{array}$$ (10)

where λ is the occurrence rate of events. The growth factor *b* may be estimated from the average of the changes of the forecast values in the data, i.e., *b* = (*x*′ − *x*)/*x* = (*z*′ − *z*)/*z*.

Suppose that there are a total of *N* forecasts at given time *t*. The error in each forecast can change according to the transition rate in Eq 10. Then the time evolution of the probability *P*(*x*_1_, …, *x*_*N*_;*t*) of the *N* forecast errors is conveniently described by a master equation [51]. In terms of the distribution function, relating with the probability *P*(*x*_1_, …, *x*_*N*_;*t*) via

$$\begin{array}{c} f\left(x,t\right)=\frac{1}{N}\int dx_{1}\cdots dx_{N}\sum_{i}\delta \left(x_{i}-x\right)P\left(x_{1},\ldots ,x_{N};t\right), \end{array}$$ (11)

the master equation reduces to the evolution equation

$$\begin{array}{c} \frac{\partial }{\partial t}f\left(x,t\right)=-\left(r+\lambda \right)f\left(x,t\right)+\frac{\lambda }{1+b}f\left(\frac{x}{1+b},t\right)+rg\left(x,t\right), \end{array}$$ (12)

where *g*(*x*, *t*) is the distribution function of newly produced forecast errors with the production rate *r*. Here production of a forecast is referred to as the issuance of the first forecast on a firm by an analyst in a given (fiscal) year.

We first consider the case that new forecast errors are produced according to the current distribution. In this case of *self-size production*, where the distribution of updated forecast errors is identical to that of existing forecast errors [*g*(*x*, *t*) = *f*(*x*, *t*)], the solution of the master equation is given by the log-normal distribution [40]:

$$\begin{array}{c} f\left(x,t\right)=\frac{1}{\sqrt{2\pi }\sigma_{t}x}\exp\left[-\frac{{\left(\ln x-\mu_{t}\right)}^{2}}{2{\sigma_{t}}^{2}}\right], \end{array}$$ (13)

where the mean and the deviation evolving in time are given by

$$\begin{array}{ccc} \mu_{t} & = & \lambda t\ln\left(1+b\right)+\mu_{0}\\ \sigma_{t} & = & \sqrt{\lambda t}\left|\ln(1+b)\right|+\sigma_{0} \end{array}$$ (14)

with the initial mean *μ*_0_ and deviation *σ*_0_.

It is remarkable that the empirical fit in Fig 1 exhibits the same form of the log-normal distribution in Eq 13. In particular, the time evolution is the same, with the identification *A* = λ ln(1+*b*) and $B=\sqrt{\lambda }|\ln(1+b)|$; this manifests that the analyst forecast indeed grows in a similar manner to the particle size growth.

Further, the power-law type distribution can also be explained with the same model. Specifically, analysts in the tail are expected to produce new (extreme) forecasts, whose value may vary within a limited range and are thus more or less uniform. In such a case of *uniform-size production*, i.e., *g*(*x*, *t*) = *δ*(*x* − *x*_0_), the stationary-state solution of Eq 12 is given by the power-law distribution [40]:

$$\begin{array}{c} f\left(x\right)=\frac{r}{\lambda bx_{0}}{\left(\frac{x}{x_{0}}\right)}^{-(\alpha +1)}\theta \left(x-x_{0}\right) \end{array}$$ (15)

with the Heaviside step function *θ*(⋅), where the power-law exponent depends on the parameters as follows:

$$\begin{array}{c} \alpha =\frac{\ln(1+r/\lambda )}{\ln(1+b)}. \end{array}$$ (16)

Note that the distribution shape of the newly produced forecasts works as a discriminating factor between the main body and the tail. A log-normal distribution can be well explained if the distribution of newly produced forecasts are almost identical to that of the existing forecasts. On the other hand, if newly produced forecasts are almost uniform, there emerges a power-law distribution. The proposed model, when appropriate parameters associated with analyst forecast behaviors are recognized, thus yields distributions for both cases.

For quantitative comparison, we use *b* = −0.12 ± 0.04, λ = (1.11 ± 0.48) × 10^−2^, and *r* = (5.5 ± 4.4) × 10^−3^, estimated from empirical data for the main body, to obtain the slopes of *μ*_*t*_ and of *σ*_*t*_: λ ln(1+*b*) = −0.0015 ± 0.0008 and $\sqrt{\lambda }|\ln(1+b)|=0.014\pm 0.006$, respectively. Within error bars, these values are reasonably consistent with the slopes *A* and *B* obtained from the empirical analysis, which confirms the relevance of our model. As for the tail part, which exhibits power-law behavior, we obtain the growth factor *b* = 0.30 ± 0.05, update rate λ = (9.3 ± 2.6) × 10^−3^, and production rate *r* = (5.4 ± 2.0) × 10^−3^, estimated from empirical data for the extreme forecasts. Using these parameters in Eq 16 leads to *α* = 1.8 ± 0.7, which, albeit demonstrating the emergence of complexity, is appreciably larger than the empirical value of unity.

To resolve this discrepancy in *α*, we extend our model to incorporating the effects of sensing other analysts’ forecasts. Such *interactions* among constituents have never been considered in the growth problem. We assume that there is a tendency toward reducing the difference from other forecasts, which leads to the contribution *c*_*ij*_(*x*_*j*_ − *x*_*i*_) of the *j*th forecast to the amount of growth of the *i*th forecast error. In case that detailed information on *c*_*ij*_ is not available, it is rational to avoid any bias. We thus take the uniform interaction strength *c*_*ij*_ = *c*/*N*, which is least biased and corresponds to the mean-field theory in physics [53], and sum over *j* to obtain $\Delta x_{i}=bx_{i}+c\bar{x}$, where *c*/*N* has been absorbed into *b* and $\bar{x}\equiv N^{-1}\sum_{j(\neq i)}x_{j}$ is essentially the average forecast error. In consequence, Eq 10 changes into

$$\begin{array}{c} \omega \left(x_{i}\to x_{i}^{\prime }\right)=\lambda \delta \left[x^{\prime }-\left(1+b\right)x_{i}-c\bar{x}\right], \end{array}$$ (17)

and the *i*th argument *x*_*i*_ of the probability *P*(*x*_1_, …, *x*_*N*_;*t*) is therefore replaced by $x_{i}-c\bar{x}$ in the master equation. In terms of ${\tilde{x}}_{i}\equiv x_{i}-c\bar{x}$, we thus have the same expression, which is to be integrated over all *x*_*i*_. This can be performed by noting $dx_{1}\ldots dx_{N}=J_{N}^{-1}d{\tilde{x}}_{1}\ldots d{\tilde{x}}_{N}$, where *J*_*N*_ is the Jacobian determinant of the mapping between *x* and $\tilde{x}$. Accordingly, the presence of interactions introduces the Jacobian determinant in the result. Although it is formidable to calculate exactly the *N* × *N* determinant, the lowest-order correction may be obtained for weak interactions (*c* < 1).

In the case of the stationary (power-law) distribution, which is our concern, Eq 16 is modified as follows:

$$\begin{array}{c} \alpha^{\text{int}}=\frac{\ln\left(1-c^{2}/2\right)+\ln\left(1+r/\lambda \right)}{\ln(1+b)}. \end{array}$$ (18)

Empirical data indeed show that analysts in the tail interact with each other. With the mean growth factor *b*, the interaction strength is defined according to $z_{i+1}-z_{i}=bz_{i}+c_{i}\left(\bar{z}-z_{i}\right)$, which leads to $c=\left[z_{i+1}-\left(1+b\right)z_{i}\right]/\left(\bar{z}-z_{i}\right)$ with the precalculated value of *b*. Taking the average, we thus estimate *c* ≈ 0.58, although large uncertainty due to strong fluctuations among analysts apparently restricts the relevance of this mean value. Putting this value into Eq 18, we obtain *α*^int^ ≈ 1.1. This value is substantially closer to the empirical value *α* ≈ 1, which is remarkable in view of the simple consideration of the interactions.

## Discussion

Financial analysts play a substantial role in financial markets as they collect, process, and disseminate valuable information to market participants, especially to retail investors who generally lack the time and resources to perform research on companies individually. Hence, whether analyst forecasts offer unbiased information in a timely manner is important. Our study focuses on the influence of information disparity on the analyst behavior: effects of 1) Regulation FD, 2) information dissemination throughout a fiscal year, and 3) interactions among financial analysts.

By dividing optimistic analyst forecast errors into extreme forecasts above the 95th percentile and the remaining non-extreme ones below, we document different analyst forecast behaviors in the two groups. The main body of forecast errors fits a log-normal distribution. On the other hand, the tail of forecast errors follows a power-law distribution with exponent *α* ≈ 1 in normal years. During recessions, there are more intensive collective behaviors among extreme analyst forecasts before Regulation FD, while we observe less collective behavior after Regulation FD.

Carrying out big-data analysis on the time-series of annual power-law exponents, we find that the power-law exponents of forecast errors are mostly close to unity, exhibiting Zipf’s law. During the years of economic recessions, the exponents deviate from unity, implying that analyst forecasts behave differently during the period of high information disparity. Within a year, the monthly power-law exponent decreases with time, indicating that less collective behavior comes into play among the extreme forecasts. Further, the cut-off point of the 95th percentile moves closer to the center towards the year-end, suggesting that more forecasts herd with the majority. On the other hand, probing the monthly pattern in non-extreme forecasts, we observe that the corresponding log-normal distribution evolves in accordance with the mean and the variation growing linearly in time and proportionally to the square root of time, respectively.

Making use of the analogy with the growth of particles, we have proposed a theoretical model for the dynamics of forecasts. It provides a good description, not only qualitatively but also quantitatively, of such features as the power-law distribution of the tail as well as the log-normal distribution of the main body, evolving through the mean and the deviation growing in time. In particular, the model, incorporating interactions between analysts, gives the power-law exponent in excellent agreement with the empirical result. We thus believe that our theoretical model, despite its simplicity, captures the key ingredients relevant to the actual dynamics of analyst forecasts.

These findings shed light on our understanding of how analysts form their forecasts, based not only on material public information but also on existing forecasts of other analysts; this influences substantially analyst forecasts. This is more distinct when the level of information disparity in the market is higher.
